# Lipid Lowering Therapy Utilization and Lipid Goal Attainment in Women

**DOI:** 10.1007/s11883-025-01275-1

**Published:** 2025-01-28

**Authors:** Julie A. E. van Oortmerssen, Janneke W. C. M. Mulder, Marte F. van der Bijl, Ruben J. M. Mijnster, Maryam Kavousi, Jeanine E. Roeters van Lennep, on behalf of the IMPRESS consortium

**Affiliations:** 1 Department of Epidemiology, Erasmus University Medical Center Rotterdam, Rotterdam, The Netherlands; 2 Department of Internal Medicine, Erasmus MC Cardiovascular Institute, University Medical Center Rotterdam, Rotterdam, The Netherlands; 3 Department of Internal Medicine, Erasmus University Medical Center Rotterdam, Dr. Molewaterplein 40, Rotterdam, 3015 GD The Netherlands

**Keywords:** Lipids, Cholesterol, Anticholesteremic agents, Lipid lowering drugs, Women, Cardiovascular diseases

## Abstract

**Purpose of Review:**

The purpose of this review is to provide an overview of the current status of lipid-lowering therapy utilization and lipid goal attainment in women. We focus on lipid-lowering therapy in individuals with and without established atherosclerotic cardiovascular disease, as well as familial hypercholesterolemia. Additionally, this review aims to explore the underlying mechanisms driving these sex differences and to identify existing knowledge gaps in this area.

**Recent Findings:**

Despite the proven efficacy of lipid-lowering therapy in both sexes, real-world studies indicate that women with comparable risk profiles are less likely than men to receive these treatments. Furthermore, women who are prescribed statins typically receive lower-intensity regimens than men and are less likely to achieve guideline-recommended low-density lipoprotein cholesterol goals.

**Summary:**

Despite advancements in lipid-lowering therapies, women compared to men, are systematically undertreated. This difference is influenced by patient-related, physician-related, and societal factors.

**Graphical Abstract:**

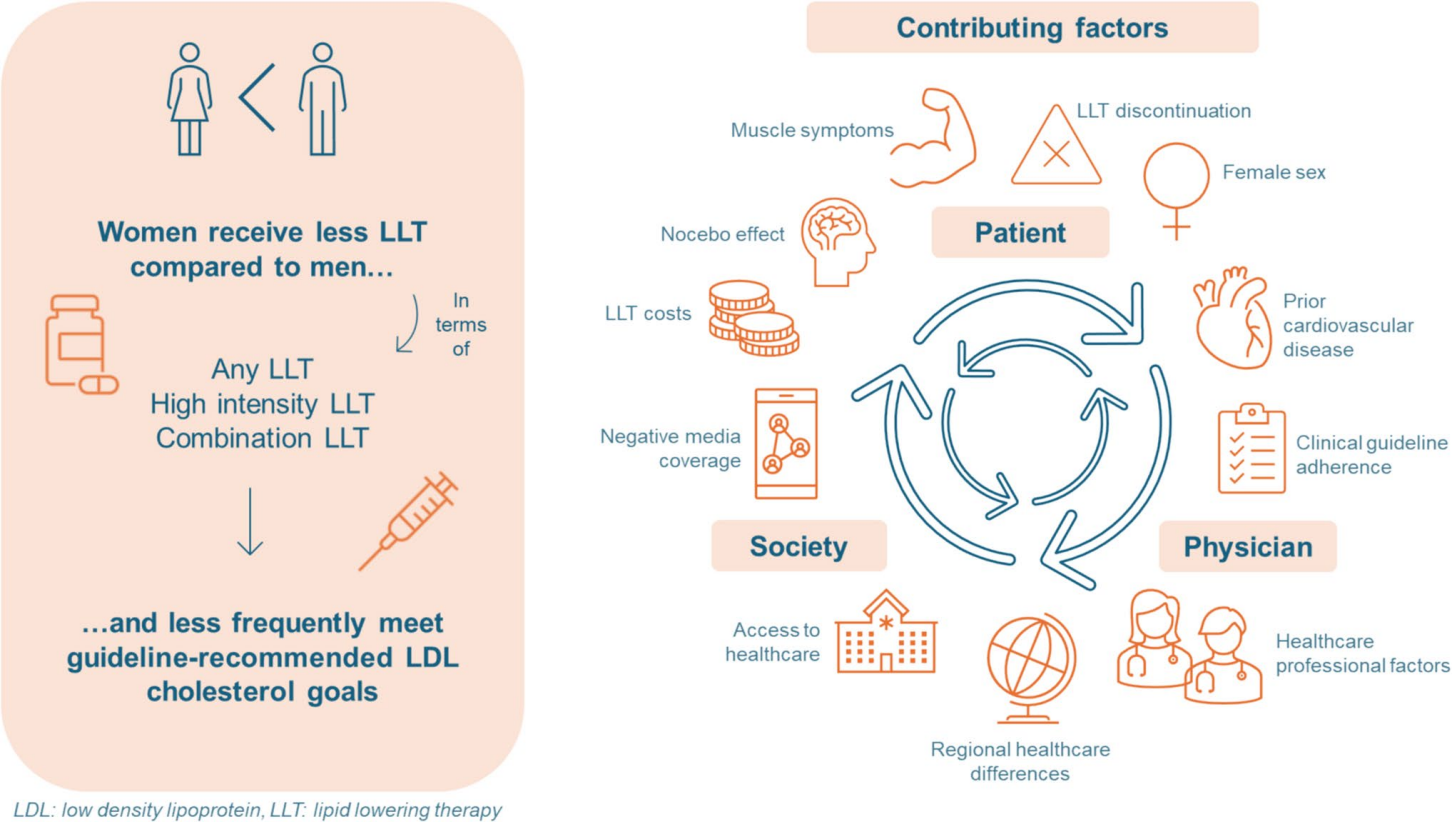

## Introduction

Atherosclerotic cardiovascular disease (ASCVD) is the leading cause of mortality worldwide and a major contributor to disability [1, 2]. Risk factors for ASCVD are largely well-established and modifiable, including diabetes, hypertension, dyslipidemia, smoking, and physical inactivity [1, 3, 4]. More than 90% of the risk for ASCVD is attributable to these factors, indicating a substantial opportunity for prevention [5]. In recent decades, landmark trials of lipid-lowering therapies (LLT), including statins, ezetimibe, and proprotein Convertase Subtilisin/Kexin type 9 (PCSK9) inhibitors, have demonstrated a reduction in both initial and recurrent ASCVD events. These findings underscore the causal role of low-density lipoprotein (LDL) cholesterol in the development of ASCVD [6].

Over the past decade, the prevalence of ischemic heart disease in young women has significantly increased due to unfavorable lifestyle changes [7]. Women continue to experience worse outcomes compared to men, with mortality from coronary artery disease notably higher in women than in men (51% versus 42%) [8–10]. This disparity in outcomes is particularly pronounced after acute myocardial infarction and percutaneous coronary interventions [11, 12]. Additionally, women with diabetes mellitus face a 50% higher risk of fatal coronary heart disease compared to men [13]. Missed or delayed diagnosis and undertreatment of ASCVD contribute significantly to morbidity and mortality [14, 15], with evidence showing that women are less likely than men to receive guideline-recommended treatment [16–21]. Sex and gender differences in ASCVD can be attributed to various factors [22]. Biological influences on ASCVD risk include hormonal changes such as premature menopause, pregnancy-related risks, and the use of oral hormonal contraceptives [23]. Gender-related factors consist of social and community factors, such as socio-economic status, patient-provider communication, and healthcare satisfaction [8].

Among cardiovascular risk factors, dyslipidemia has the highest population-adjusted risk in women, at 47.1% [21]. Since LDL cholesterol is a major modifiable risk factor for which effective and safe treatment options are available, it is important to assess sex differences in LLT utilization as well as cardiovascular outcomes [24, 25]. In this review, we will provide a comprehensive overview of the current literature regarding advances in hypercholesterolemia research, the diverse treatment options available to address this preventable yet persistent contributor to ASCVD, and the attainment of LDL cholesterol goals in women.

## ASCVD Prevention

### No established ASCVD

LDL cholesterol plays a direct causal role in the development of ASCVD. Lowering LDL cholesterol is widely recognized as a critical therapeutic approach to mitigate ASCVD risk, particularly among those at highest risk of future cardiovascular events [24, 26–29]. LLT, particularly statins, is universally recommended for the prevention of ASCVD [28, 29]. Statins reduce the risk of major cardiovascular events by approximately 20% for each 1 mmol/L reduction in LDL cholesterol concentration across various populations, with equal effectiveness observed in both women and men [30]. Therefore, current ASCVD prevention guidelines offer similar recommendations in women and men. However, several real-world studies showed that women are less likely than men with similar ASCVD risk to be prescribed statin therapy [17, 18, 31–36]. Moreover, among those prescribed a statin, women typically receive lower intensity regimens compared to men and consequently less frequently achieve guideline-recommended LDL cholesterol goals [17, 32, 37–43]. For example, in the PORTRAIT-DYS study among 30,323 Portuguese individuals aged 40–85, women were 22% less likely to achieve ESC/EAS LDL cholesterol goals (HR 0.78, 95%CI 0.73–0.82) [37]. Despite the documented efficacy of statin therapy in both sexes, residual doubt of its effectiveness in women without established ASCVD may partially explain the differences in statin intensity use [32, 44–46]. Contradictory results may have created doubt about the benefits of statins for women at low ASCVD risk. Concerns regarding the effectiveness of statins in women without ASCVD compared with men stemmed primarily from historical underrepresentation in clinical trials, inconsistent results from sex-specific analyses, a higher incidence of adverse effects, and adherence challenges among women. However, more recent evidence, including large meta-analyses and findings from the JUPITER trial, supports the efficacy of statins in reducing cardiovascular events in women, comparable to the benefits observed in men [47]. General practitioners are also hesitant to prescribe high-intensity statins to women due to concerns about adverse events and potential impacts on adherence, as high-intensity statin therapy has been associated with lower adherence, especially in women [45, 48]. Multiple studies suggest women are at higher risk of reporting statin-related adverse events and are more likely to discontinue statins, compared to men [17, 44, 45, 49, 50].

The value of statin therapy in cardiovascular risk prevention in the elderly (> 75 years of age) remains less certain, necessitating a tailored approach for every patient after discussing the risks and benefits [7]. Cardiovascular primary prevention guidelines suggest that statin therapy is recommended in elderly patients who are expected to derive clinical benefit from treatment [7, 51]. A meta-analysis showed that initiating statin therapy was significantly associated with reductions in major cardiovascular events, including cardiovascular mortality and all-cause mortality [52, 53]. However, potential side effects of statins are of concern in the elderly due to multimorbidity, polypharmacy, and frailty. Additionally, since statins require an extended period of use to show benefits, patient life expectancy should also be considered in the overall decision-making process for statin therapy [5, 51].

### Established ASCVD

The Scandinavian Simvastatin Survival Study (~ 18–19% women) was the first of many placebo-controlled randomized clinical trial to demonstrate reductions in cardiovascular events and improved survival in patients with established ASCVD [54–56]. The IMPROVE-IT trial (~ 25% women) showed that adding ezetimibe to statins further reduced LDL cholesterol and achieved a 6.7% relative risk reduction in cardiovascular outcomes over 7 years for patients with acute coronary syndrome, offering a therapeutic option for those who insufficiently reach guideline-recommended LDL cholesterol goals with statins alone [57]. Similar to individuals without established ASCVD, women are less likely than men to receive guideline-indicated LLT [58–62].

More recently, PCSK9 inhibitors monoclonal antibodies (mAbs) have become an additional LLT option, with alirocumab and evolocumab achieving an average LDL cholesterol reduction of 55–60%, leading to an improvement in cardiovascular outcomes [63–68]. Both PCSK9 mAbs have a favorable safety profile and no sex differences were observed in their association with cardiovascular endpoints [63, 64, 67, 69]. Sex-specific analyses of clinical trials and some real-world studies have shown that women experience a slightly lesser relative reduction in LDL cholesterol with PCSK9 mAbs compared to men [70–74]. For instance, a meta-analysis of 16 trials reported a mean reduction of 47.5% in women versus 54.1% in men at 24 weeks [75]. Although the absolute reduction in LDL cholesterol is comparable between women and men, higher baseline LDL cholesterol levels in women result in a lower proportion reaching target LDL cholesterol levels [72, 74]. This disparity in relative LDL cholesterol reduction between sexes may be attributed to sex differences in circulating levels of PCSK9 [76, 77]. Several studies have indicated that women have higher circulating PCSK9 concentrations than men [76]. PCSK9 levels fluctuate with the menstrual cycle and with menopause status, they are presumed to be influenced by estrogen, possibly through G-protein coupled estrogen receptor activation on hepatocytes [77, 78]. Due to the decrease of estrogen, especially post-menopause, women’s PCSK9 concentrations increase, resulting in increased LDL cholesterol levels [79].

### Familial Hypercholesterolemia

Familial hypercholesterolemia (FH) is a common condition occurring approximately 1:313, caused by pathogenic variants in the *LDLR*,* PCSK9*, or *APOB* gene affecting LDL metabolism [80]. Persons with FH have elevated LDL cholesterol levels from birth onwards and consequently an elevated risk of premature ASCVD. ASCVD risk can be greatly reduced by starting LLT with statins and if needed combination therapy [81, 82].

Various real-world studies have observed that women with FH are less likely to receive LLT. A recent meta-analysis of > 129,000 patients with FH showing that women had significantly lower odds of using LLT (OR of 0.74 (95% CI 0.66–0.85)) [83]. This sex difference in LLT utilization was consistent across all world regions studied, and did not show an improvement over time. Moreover, when women with FH are treated with LLT, they receive less intensive and less combination LLT compared to men [83–85]. Unsurprisingly, this led to less women with FH achieving guideline-recommended LDL cholesterol goals compared to men (OR 0.74 (95% CI 0.66–0.85)) [83].

On the other hand, other studies found no sex differences in the use of LLT, such as the Spanish SAFEHEART registry, consisting of 3,506 women and men with FH [86]. However, they did note sex differences at enrollment, with a lower proportion of women receiving high-intensity statins (46.9% vs. 53.6%, *p* < 0.01) and combination LLT (44.4% vs. 56.7%, *p* < 0.01) [86]. Some studies report no sex differences in LDL cholesterol goal attainment in persons with FH [87, 88], suggesting potential differences in management between treating healthcare professional practices.

The most severe and rarest form of FH is homozygous FH (HoFH), which is caused by biallelic pathogenic mutations in the *LDLR*,* PCSK9*,* APOB or LDLRAP1* gene [89]. The prevalence of HoFH is approximately 1:360,000. Persons with HoFH have extremely elevated LDL cholesterol levels leading to exceptionally premature ASCVD or death, often before the age of 20 years. Treatment includes the aforementioned LLTs, along with additional interventions such as lipoprotein apheresis, MTTP inhibitor lomitapide, and the ANGPTL3 monoclonal antibody evinacumab [89]. The largest international registry of 751 patients with homozygous FH from 38 countries observed no sex differences in the type or number LLT used or in the attainment of LDL cholesterol goals [90]. Both women and men with homozygous FH rarely achieve guideline-recommended LDL cholesterol goals (4.0% and 3.1%, *p* = 0.82, respectively).

## Lipid Goal Attainment

### Side Effects of LLT

Although statins are generally well tolerated, statin intolerance is common in clinical practice and is frequently cited as a reason for discontinuation or modification of statin therapy [91, 92]. This has important clinical implications since poor adherence to or cessation of statin therapy is associated with higher risk for adverse cardiovascular outcomes, but also increased healthcare costs [93–97]. Statin intolerance is defined as adverse effects from statin therapy that improve or resolve with dose reduction or discontinuation. Diagnosis requires that at least two different types of statins have been tried, including one at the lowest approved dose [98]. The most frequently reported statin side effects are statin-associated muscle symptoms, with an incidence ranging from 5% in clinical trials to 20% in observational studies [91, 99, 100]. Less commonly, statin therapy is associated with myopathy (1 in 10,000 per year) and rhabdomyolysis (1 in 100,000 per year) [91].

Women are more likely than men to discontinue or switch statin therapy due to the onset or worsening of muscle symptoms [100–102]. This increased susceptibility to statin-associated muscle symptoms in women may be influenced by various factors. It is important to realize that women report more adverse events in all circumstances as was shown by a meta-analysis of six atorvastatin trials, which revealed that women discontinued study medication more often than men, regardless of whether they were randomized to placebo or atorvastatin. However, a treatment-by-gender interaction was only present in one of the six trials [45]. These findings suggest that biological differences between sexes may not fully explain these variations. However, certain biological sex differences could account for the higher prevalence of statin-related side effects in women. Differences in body composition, such as higher body fat and lower muscle mass in women, may lead to higher statin concentrations and an increased risk of adverse effects [103, 104], but also genetic factors may contribute to statin-induced myopathy. A single-nucleotide polymorphism in SLOC1B1 on chromosome 12, which encodes the organic anion-transporting polypeptide 1B1 responsible for hepatic statin uptake, may have a greater impact on women than men [105, 106]. Moreover, women exhibit greater sensitivity to pain, which may contribute to the higher incidence of muscle symptoms [107].

Interestingly, a growing body of evidence suggests that the nocebo effect plays a significant role in statin intolerance, where expectations of adverse effects lead patients to attribute muscle symptoms to statins [108–111]. Previous studies indicate that 80–90% of the symptom burden may be explained by this effect [108–110]. Female sex also appears to be a risk factor for the nocebo effect in some but not all studies [112].

PCSK9 inhibitors showed no significant differences in safety profiles between women and men, except in the FOURIER trial, where women compared to men experienced more side effects and injection site reactions with evolocumab [69, 113–115]. However, this difference was not confirmed in recent observational studies [72, 74]. Overall, there is little evidence to suggest that the safety profile of PCSK9 inhibitors differs between women and men.

### Possible Factors Influencing (Sex) Differences in LLT Use

Several factors may contribute to sex differences in LLT utilization, including patient preferences, healthcare professional practices, and societal influences. For instance, a retrospective cohort study in a primary care setting of 24,212 patients with ASCVD not using statins found that women were more likely than men to decline statin treatment (24.1% versus 19.7%) [116]. Similar findings were observed in a nationwide registry study of 5,693 patients at risk of or with ASCVD [17]. Additionally, female gender has been associated with a greater concerns about side effects [117, 118], and women are more likely to discontinue or be less adherent to statin therapy [17, 102, 119, 120].

Healthcare professional factors may also contribute to the sex differences in LLT utilization. Women have their lipid levels measured less frequently in general practice [59], and they are less likely to be referred to or seen by a medical specialist [121]. Additionally, studies have observed differences related to medical specialty, with general practitioners being less likely to prescribe optimal statin therapy compared to medical specialists [122, 123]. Misperceptions by healthcare professionals regarding patients’ LDL cholesterol attainment rates or cardiovascular risk may also contribute to lower LLT utilization [124], particularly since these misperceptions appear to be more common concerning female patients [122]. In a nationwide registry study of 5,693 patients at risk or with ASCVD, a greater proportion of women than men reported not having been offered statin therapy [17]. Furthermore, a US study employing a deep learning approach to analyze clinical note discussions of statin prescriptions found that, among patients not using statins, women with ASCVD were less likely than men to be prescribed statins or high-intensity doses when prescribed, with greater disparities observed among younger patients, those with private insurance, and English speakers [42]. Additionally, among individuals not prescribed statins, women were less likely to have statins mentioned in clinical notes and were slightly more likely to have documented statin intolerance [42].

Societal factors that may influence the uptake and utilization of LLT include variations in insurance coverage [123, 125] and out-of-pocket costs for medication [126, 127]. Regional differences in lipid goal attainment between sexes have been observed, with women in Europe having lower odds than men of achieving treatment targets for total cholesterol and LDL cholesterol [128]. These disparities were smallest in Europe and largest in the Middle East, where women had the lowest odds of reaching these goals compared with men [128]. Additionally, a study in the United States found that geographic region strongly correlated with high-intensity statin use after ASCVD, contributing to substantial treatment disparities [129]. Furthermore, negative media-coverage and public discussions about statins impact statin uptake and continuation, with negative portrayals leading to higher discontinuation rates [96, 117, 130–132].

### Clinical Trial Participation

Women are still significantly underrepresented in cardiovascular drug trials, particularly for heart failure, coronary artery disease, and acute coronary syndrome, with an average participation-to-prevalence ratios < 0.8 [133–135]. A systematic review of 60 randomized controlled trials on LLT published between 1990 and 2018 found that only 28.5% of participants were women, whereas the background population consisted of an equal number of women and men [136]. Although the enrollment of women in LLT trials increased from 19.5% in 1990–1994 to 33.6% in 2015–2018, women remained underrepresented relative to their disease burden. Additionally, only 53.0% of these trials reported outcomes by sex, with no significant improvement over time [136]. This underrepresentation of women in clinical trials raises significant concerns. Biased enrollment practices can undermine trust in the medical and scientific communities and lead to inequitable access to potential health benefits for trial participants [137]. Moreover, individual trials often lack the statistical power to detect sex differences in outcomes. Inclusion of a more diverse population is important, enabling meta-analyses to identify potential differences in treatment efficacy and safety between sexes [137]. Given known sex-based differences in the absorption and distribution of therapeutic agents, balanced representation in trials and the reporting of sex-specific outcomes are important for future research [138].

Despite numerous hypotheses regarding women’s underrepresentation in LLT interventions, evidence remains limited. A review of 59 large LLT trials published between 1994 and 2018 revealed that women comprised less than 20% of the authorship, with no change over time [139]. A more recent cross-sectional study examined the proportion of women as principal investigator in trials listed on ClinicalTrials.gov between January 2005 and May 2023 and found that the proportion of women principal investigators was the lowest (20.3%) in the field of cardiology [140]. This contrasts with trends in other medical fields, where female representation has increased [141, 142]. Notably, women may be more likely to participate in trials led by female investigators, who may also design studies that are more inclusive of female participants [143]. Additionally, a recent scoping review underscores the critical role of study design in addressing the underrepresentation of women in cardiovascular clinical trials [144]. Barriers to participation include concerns about randomization, insufficiently engaging information, and practical challenges such as financial costs, time constraints, and caregiving responsibilities. Additionally, women’s willingness to participate is influenced by their risk perception and limited recognition of CVD as a leading cause of death [14, 145]. Exclusions due to age limits, multi-morbidity, or other sex-specific factors (for example, pregnancy, contraception use, menopause) may further limit women’s participation [144]. Enhancing participation may involve providing sex-specific trial information, facilitating discussions with healthcare providers or past participants, and addressing logistical barriers through flexible scheduling, childcare support, and financial reimbursement [144].

## Conclusions

Significant advancements in LLT have markedly reduced ASCVD risk in recent years. However, several critical questions remain, necessitating further research to refine both primary and secondary ASCVD prevention strategies. One key area requiring investigation is the evident sex differences in LLT utilization, discontinuation rates, and failure to achieve guideline-recommended LDL cholesterol goals. Future studies should focus on understanding why women are less likely to be prescribed or accept statin therapy, determining optimal sex-specific statin regimens, and developing strategies to improve LDL cholesterol goal attainment and adherence. Research is also needed to explore the nocebo effect’s role in statin intolerance and whether female sex is a risk factor. Additionally, investigations should examine whether provider- and organization-level factors contribute to the observed sex and gender differences in statin use. Both qualitative and quantitative studies, as well as mixed-methods studies, may be particularly valuable in uncovering these underlying factors. Further research is warranted to understand the mechanisms behind sex differences in PCSK9 levels, which could clarify the differential effects of PCSK9 inhibitors on relative LDL reduction between women and men. Moreover, addressing the underrepresentation of women in cardiovascular disease and LLT trials is paramount. Identifying and overcoming barriers to female participation will ensure more inclusive study results. In all proposed investigations, it is essential to consider differences across various subgroups, including sex, ethnicity, and individuals with FH.

## Key References

- Roeters van Lennep JE, Tokgozoglu LS, Badimon L, Dumanski SM, Gulati M, Hess CN, et al. Women, lipids, and atherosclerotic cardiovascular disease: a call to action from the European Atherosclerosis Society. Eur Heart J. 2023;44(39):4157-73.
  - ○ Position statement from the European Atherosclerosis Society on sex-specific cardiovascular risk factors and their impact on lipids and cardiovascular risk throughout the life course.
- Iatan I, Akioyamen LE, Ruel I, Guerin A, Hales L, Coutinho T, Brunham LR, Genest J. Sex differences in treatment of familial hypercholesterolaemia: a meta-analysis. Eur Heart J. 2024 Sep 14;45(35):3231–3250.
  - ○ Meta-analysis on sex differences in familial hypercholesterolemia care: use of lipid-lowering treatment, treated LDL cholesterol levels, and lipid goal achievement.

## Data Availability

No datasets were generated or analysed during the current study.
